# Identification of A p300–SP1–BRD4 Transcriptional Axis as a Key Driver of AR Hyperactivation in Polycystic Ovarian Syndrome

**DOI:** 10.1002/advs.202518185

**Published:** 2026-02-13

**Authors:** Zhengquan Zhu, Yihan Wang, Haiyun Chen, Xinye Yu, Tingyu Wang, Yajing Weng, Meihong Guo, Ying Huang, Gaojian Tao, Wangsen Cao, Yong Wang, Daojuan Wang

**Affiliations:** ^1^ Department of Pain Management Nanjing Drum Tower Hospital Affiliated Hospital of Medical School Nanjing University Nanjing China; ^2^ Department of State Key Laboratory of Analytical Chemistry For Life Science and Jiangsu Key Laboratory of Molecular Medicine Medical School of Nanjing University Nanjing China; ^3^ Department of Nephrology Yangzhou Precision Research Institute of Kidney Disease Northern Jiangsu People's Hospital Teaching Hospital of Nanjing University Medical School Yangzhou China

**Keywords:** acetylation modification, androgen receptor, p300, PCOS, SP1

## Abstract

Persistent androgen receptor (AR) activation is an important contributor to polycystic ovary syndrome (PCOS) and is affected by transcriptional regulation via histone acetylation; however, the underlying mechanisms are partially understood. This study demonstrated that AR activation in ovarian granulosa cells (GCs) of both dehydroepiandrosterone (DHEA) and high‐fat diet‐induced PCOS mouse models correlated with a significant increase in the histone acetyltransferase p300 and histone acetylation. Conversely, GC‐specific p300 knockout or pharmacological inhibition with C646/A‐485 effectively reduced AR activation, histone 3 acetylation (H3K18ac/H3K27ac), and ovarian fibrosis in PCOS mice, highlighting p300 as a critical driver. ATAC‐seq and RNA‐seq identified “open” chromatin regions at the AR promoter in PCOS ovaries, corresponding with increased AR transcription and histone acetylation. p300, along with transcription factor SP1 and the acetyl‐reader BRD4, bound to H3K18ac and H3K27ac of the AR promoter in PCOS‐modeled ovaries and GCs, which was blocked by C646 and the SP1 inhibitor Plicamycin, respectively. Importantly, continuous AR activation by its ligand DHT largely diminished the anti‐fibrotic and ovarian‐protective effects of C646. These findings suggest that p300, SP1, and BRD4, form a critical transcriptional complex driving AR activation and PCOS development, and that targeting the p300/AR axis may present a promising therapeutic approach for treating PCOS.

## Graphical abstract

This study reveals a pivotal epigenetic regulatory role for the histone acetyltransferase p300, demonstrating that its acetylation of histone 3 at lysine 18 and 27 (H3K18ac and H3K27ac), alongside the formation of the p300/BRD4/SP1 complex, drives AR activation and ovarian fibrosis in PCOS. Both granulosa cell‐specific ablation of p300 and pharmacological blockage with A‐485 or C646 block the process.

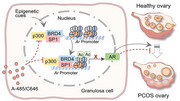

## 1 Introduction

Polycystic ovary syndrome (PCOS) is the most common endocrine and metabolic disorder, affecting an estimated 8–13% of women of reproductive age worldwide [1, 2]. Pathologically, PCOS is characterized by an increase in antral follicles and ovarian stroma, abnormal granulosa cell development, impaired follicle formation, and ovulatory dysfunction, often associated with hyperandrogenism [3, 4, 5]. Granulosa cells (GCs), the most abundant somatic cells in the follicle, are essential in follicle development and oocyte growth by providing nourishment. They also contribute to the rupture of the follicular wall and the release of the oocyte in response to follicle‐stimulating hormone (FSH) and luteinizing hormone (LH) [6, 7]. Past studies have shown that persistent activation of the androgen receptor (AR) in GCs, even in the absence of elevated androgen levels, plays a central role in the development of PCOS. This is driven by mechanisms such as oxidative stress, inflammation, increased TGF‐β signaling, collagen synthesis, and ovarian fibrosis, all of which hinder follicular development [8, 9, 10]. However, the precise upstream regulatory mechanisms involved in these processes remain poorly understood.

The AR is a nuclear receptor and transcription factor consisting of three functional domains: a highly variable N‐terminal domain (NTD), a conserved DNA‐binding domain (DBD), and a C‐terminal ligand‐binding domain (LBD) [11, 12, 13]. AR abnormalities have been implicated in various diseases, including androgen insensitivity syndrome, benign prostatic hyperplasia, prostate cancer, and an increased risk of cardiovascular disease [14, 15, 16, 17, 18]. In addition to being activated by androgens, AR activity is regulated by transcriptional and post‐transcriptional mechanisms, which involve both non‐epigenetic and epigenetic processes [19], such as DNA methylation [20, 21], non‐coding small RNAs [22], and protein/histone acetylation. For example, AR suppression due to aberrant DNA methylation patterns in the *AR* gene [23] and miRNA targeting the *AR* mRNA [24, 25] is the main cause of androgen insensitivity and resistance to anti‐androgen therapy. Our previous study demonstrated that the chromatin acetylation “reader” BRD4, along with HIF‐1α, bound to acetylated histones at the *Ar* promoter in PCOS ovaries, thereby transcriptionally activating AR and promoting PCOS pathogenesis in rats [26]. This provides direct evidence that histone acetylation is a crucial pathological trigger for AR activation in PCOS.

Histone acetylation is regulated by histone acetyltransferases (HATs) and histone deacetylases (HDACs), which modulate the acetylation of lysine residues [26, 27, 28]. HATs‐mediated acetylation of histones neutralizes their positive charge, facilitating chromatin remodeling and creating an open conformation that promotes the downstream gene transcription [29, 30]. In contrast, HDACs primarily regulate gene expression by removing acetyl groups from histones, leading to chromatin condensation and transcriptional repression. However, HDACs also play critical roles in maintaining constitutive gene expression and preserving chromatin architecture through complex interactions with transcriptional machinery and non‐histone proteins [31]. HATs lack inherent target specificity and often work with other transcriptional cofactors to form complexes that mediate transcriptional activity. There are five main families of HATs: p300/CBP, Gcn5, MYST (MOZ, Tip60, Ybf2/Sas3, Sas2), TAFII250, and nuclear hormone‐regulated HATs (such as SRC1 and SRC3), with p300 being the most extensively studied [32, 33, 34]. Abnormal p300 activity has been linked to various pathological conditions, including cardiovascular diseases, prostate and breast cancers, and organ fibrosis [27, 35, 36, 37, 38]; however, its critical role and key targets in PCOS are yet to be fully elucidated.

In this study, we investigate the role of HATs, particularly p300, in regulating AR transcription through epigenetic mechanisms in two PCOS mouse models. We found that AR upregulation in granulosa cells is associated with increased p300 expression in PCOS ovaries. To test whether p300 directly contributes to AR activation, we employed both pharmacological inhibition and a granulosa cell‐specific *Ep300* knockout mouse model. Additionally, we used ATAC‐seq and RNA‐seq to assess changes in chromatin accessibility linked to AR activation. Our goal is to identify gene‐targeted strategies for the prevention or treatment of PCOS.

## 2 Materials and Methods

### 2.1 Animal Study

Wild‐type female C57BL/6J mice were obtained from Nanjing Junke Biotechnology Corporation (Nanjing, China). *Ep300*‐flox mice (*Ep300* ^fl/fl^, C57BL/6Cya, S‐CKO‐10576, purchased from Cyagen Biosciences Inc.) containing loxP sites flanking the second exon of *Ep300* were bred for two generations with granulosa cell‐specific Cre driver mice, *Cyp19a1*‐*Ires*‐*Cre* (*Cyp19a1*‐Cre, *C57BL/6Cya*, C001382, Cyagen Biosciences Inc.). This breeding strategy generated granulosa cell‐specific *Ep300* knockout mice (*Cyp19a1*‐*Cre*, *Ep300* ^fl/fl^, referred as *Ep300^GC^* ^‐/−^). Mouse genotype was confirmed by polymerase chain reaction (PCR) with mouse tail DNA using the following primers: *Ep300*‐flox F1: *5’‐CGCTCCATTCCGTCTCTATTCT‐3’*; *Ep300*‐flox R1: *5’‐TGCTCCGAAAGCCATAGGTTACT‐3’*; *Cre*F2: *5’‐TACACTTTTGAGACGATTCCAGGT‐3’*; *Cre*R2: *5’‐CTAGGAATGCTCGTCAAGAAGACAG‐3’*; All mice were housed in a specific pathogen‐free (SPF) facility with a 12‐h light/dark cycle conditions at 24°C, with ad libitum access to water and food. All animal procedures of the animal experiments and clinical data were approved by the Ethics Committee and the Institutional Animal Care and Use Committee of Nanjing Drum Tower Hospital, Nanjing University Medical School (2024AE01082).

Mouse models of PCOS were established by subcutaneous implantation of silicone tubing (1 cm) for sustained dehydroepiandrosterone (DHEA) for 35 days [10] or high‐fat diet feeding (60% fat) for 3 months [39]. Female mice aged 3–4 weeks were randomly assigned to following six experimental groups: (1) Sham control; (2) C646 (10 mg/kg, HY‐13823, MCE, USA, daily intraperitoneal injection for 10 days) (3) dihydrotestosterone (DHT, 1.66 mg/kg, D413176, Aladdin, China, daily intraperitoneal injection for 7 days); (4) PCOS mice: DHEA (35 days) or high‐fat diet (HF, 3 months); (5) PCOS mice treated with C646; and (6) PCOS mice treated with DHT. At the end of treatment, mice were euthanized via intraperitoneal injection of sodium pentobarbitone (50 mg/kg). Ovaries were collected and stored at ‐80°C or fixed in 4% paraformaldehyde for further analysis. At least ten animals were included in each group to obtain enough ovary tissues for all experiments. No animals were excluded from experiments unless the presence of technical issues occurred.

### 2.2 RNA Sequencing (RNA‐seq)

Total RNA was extracted from mouse ovaries using TRIzol reagent (Invitrogen, Carlsbad, CA, USA) following the manufacturer's protocol. RNA sequencing was conducted by KaiTai biotechnology Co., Ltd. (Hangzhou, China) using the Illumina Novaseq Xplus platform. Clean reads are aligned to the mouse reference genome using HISAT2 software (v2.2.1, http://daehwankimlab.github.io/hisat2/) with default settings. Aligned reads were processed into sorted BAM files with SAMtools (v1.16.1, http://github.com/samtools/). Gene expression was normalized using FPKM values calculated from gene length and total mapped reads. Differentially expressed genes (DEGs) were identified using edgeR with criteria of |log2FC| > 1 and an adjusted *p*‐value (padj) < 0.05. Data visualization and statistical plots were generated using R‐based tools.

### 2.3 Assay for Transposase Accessible Chromatin with High‐Throughput Sequencing (ATAC‐seq)

Intact nuclei were isolated from fresh ovary tissues using a lysis buffer containing nonionic detergents to remove cytoplasmic components, followed by centrifugation. Between 50000 and 100000 nuclei were used to ensure a high signal‐to‐noise ratio. Transposition was carried out by incubating the nuclei with Tn5 transposase preloaded with sequencing adapters at 37°C for 30 min. Adaptor‐ligated DNA fragements were purified using magnetic beads, amplified using limited‐cycle PCR (5–12 cycles) to prevent over‐amplification. Library quality and size distribution were assessed with an Agilent Bioanalyzer, and qPCR was used to confirm fragment sizes (∼ 100 to 1,000 bp, peaking near 200 bp for nucleosome‐free regions). Sequencing was performed on an Illumina platform by KaiTai Biotechnology Co., Ltd.

Raw sequencing data were processed using *fastp* (v0.23.4) to remove adapter‐contaminated reads (reads containing > 5 bp of adapter sequence), low‐quality reads (low‐quality reads with > 30% of bases with Phred quality score < 15), and reads with > 5% undetermined bases (N). Clean reads were aligned to the reference genome with Bowtie2 (v2.5.3). Only uniquely mapped paired‐end reads (MAPQ ≥ 30) were retained. Duplicated reads were removed using Picard MarkDuplicates (v3.1.1), and mitochondrial reads were excluded using SAMtools (v1.19.2). Tn5 transposase bias was corrected using deepTools’ alignmentSieve (v3.5.5). Genome‐wide open chromatin regions (peaks) were then identified using MACS3 (v3.0.1). Peak annotations and genomic distribution (e.g., promoters, UTRs, exons, introns, distal intergenic regions) were analyzed using custom R scripts.

### 2.4 RNA‐seq Data of Clinical Samples Acquisition and Analysis

RNA‐sequencing data of clinical human granulosa cells were obtained from the Gene Expression Omnibus (GEO) database using the search terms “human,” “PCOS,” “granulosa cells,” “ovary,” and “RNA‐seq.” Inclusion criteria required that the datasets contain RNA‐seq data from granulosa cells of women with PCOS and healthy controls. Based on these criteria, four datasets were selected: GSE293353, GSE277906, GSE155489, and GSE138518, comprising granulosa cell transcriptomic data from 15 women with PCOS and 15 healthy controls. An expression matrix was constructed, and batch effects were removed using the ComBat function from the “sva” package in R. Differential gene expression analysis was performed using the “DESeq2.” Genes with an absolute log_2_ fold change (|log_2_FC|) > 0.5 and an adjusted *p*‐value (padj) < 0.05 were considered differentially expressed genes (DEGs).

Similarly, RNA‐sequencing data for mouse ovarian tissues were retrived from the GEO database using the search terms “mouse,” “PCOS,” “ovary,” and “RNA‐seq”. Two sets were selected: GSE236248 and PRJNA1255374, comprising ovarian transcriptomic data from 6 PCOS and 6 control mice. An expression matrix was generated, and batch effects were corrected using the ComBat function (sva.). DEGs were identified using the “DESeq2” package in R with the same criteria of |log_2_FC| > 0.5 and padj < 0.05.

### 2.5 Isolation and Culture of Granulosa Cells (GCs)

Three‐week‐old C57BL/6J female mice were injected intraperitoneally with 20 IU of pregnant mare serum gonadotropin (PMSG). After 48 h, mice were euthanized by cervical dislocation, and ovaries were collected. Follicles were isolated in DMEM‐F12 medium and punctured with micro forceps to release GCs. Cell suspension was filtered through a 70 µm cell strainer to remove debris. GCs were resuspended in complete DMEM/F12 medium, plated, and cultured at 37°C in a humidified incubator with 5% CO2 and 95% air.

### 2.6 Cell Culture and Treatment

HEK293T cells were maintained in complete DMEM medium, and the human granulosa‐like tumor cell line (KGN) and primary GCs were maintained in complete DMEM/F12 medium. A PCOS cell model was established by treating cells with DHEA (25 um, 48 h). Treatments include the p300‐selective inhibitors C646 (10 um, HY‐13823, MCE, USA) and A‐485 (0, 0.1, 0.5, 1, 5, 10 um, HY‐107455, MCE, USA), p300 agonist CTPB (5 um, HY‐124960, MCE, USA), and the SP1‐selective inhibitor Plicamycin (Plica, 100 nm, HY‐A0122).

### 2.7 Plasmid Construction and Cell Transfection

The overexpression plasmid of SP1 (GV657‐BamHI/Kpnl‐SP1‐HA) was provided by Corues Biotech, China. The human *AR* promoter luciferase reporter plasmid (GV238‐Kpnl/Xhol‐hARp‐Luc) was also obtained from GeneChem Company, China. A mutant AR‐luc construct, in which the SP1 binding site (GGGGAGGGG) was mutated to TAATGTAAT, was generated by PCR‐based site‐directed mutagenesis. HEK293T cells were transfected using GenJet Plus In Vitro DNA Transfection Reagent (SL100499, SignaGen, USA) following the manufacturer's instructions

### 2.8 Estrous Cycle Monitoring

Starting two days before C646 administration, daily vaginal smears were collected from all mice over a 12‐day period. Smears were stained with toluidine blue for 30 min and examined under an optical microscope (Leica Microsystems, Germany) to assess cell morphology and determine the estrous cycles.

### 2.9 Hematoxylin‐eosin (H&E) and Masson Trichrome Staining

Ovarian tissues were fixed in 10% buffered formalin for 48 h, then paraffin‐embedded and sectioned. Sections were deparaffinized, rehydrated, and stained with hematoxylin and eosin (H&E) to evaluate tissue morphology and histopathological changes. Masson's trichrome staining was performed on ovarian sections to assess collagen deposition. All stained slides were analyzed using an optical microscope (Leica Microsystems, Germany).

### 2.10 Immunohistochemistry (IHC) Staining

Paraffin‐embedded ovarian tissue sections were incubated overnight at 4°C with specific primary antibodies against p300 (AF20080, Aifang Biological, China) and AR (ab52615, Abcam, UK). After washing, sections were incubated with HRP‐conjugated goat anti‐rabbit IgG secondary antibody at 37°C for 1.5 h. Staining was developed with diaminobenzidine (DAB) for 5 min, followed by hematoxylin counterstaining. Images were captured using an optical microscope (Leica Microsystems, Germany).

### 2.11 Immunofluorescence (IF) Staining

Immunofluorescence staining was performed as previously described [10]. Ovarian sections and GCs were incubated with primary antibodies against AR (ab52615, Abcam, UK), p300 (ab275378, Abcam, UK), BRD4 (ER1901‐02, HUABIO, China), and SP1 (A19649, ABclonal, China) following manufacturer instructions. Images were acquired using an Olympus laser scanning confocal microscope (FV3000). The results were analyzed using Fiji software. For ovarian sections, all image channels were processed with ‘Auto Threshold’ and ‘Convert to Mask’, followed by Image Calculator and obtaining colocolizated mask. For each antibody marker, the percentage of positive signal and the co‐localized area, normalized to DAPI signal, were recorded and presented as a plot. For GCs, we used Fiji software to acquire the positive Gray_Value for each antibody marker. GraphPad was then employed to plot these as peak graphs, with greater peak overlap among the three markers signifying stronger colocalization.

### 2.12 Western Blotting

Western blotting was conducted on ovarian tissue homogenates and cultured‐cell lysates essentially as before [10]. Primary antibodies used included: p300 (ab275378, Abcam), BRD4 (83375S, CST, USA), AR (ab52615, Abcam), Histone3 lysine site 9 acetylated antibody (H3K9ac, ab4441, abcam), H3K14ac (HY‐P80167, MCE), H3K18ac (13998T, CST), H3K27ac (8173T, CST), H4K5ac (HY‐P80182, MCE), H4K8ac (HY‐P80180, MCE), H4K12ac (ab46983, abcam), H4K16ac (HY‐P80179, MCE), Histone3 (H3, 17168‐1‐AP, Proteintech), Histone4 (H4, 16047‐1‐AP, Proteintech), α‐SMA (BS70000, Bioworld), CBP (AG1691, Beyotime), Gcn5 (PA002657, CUSBIO), Tip60 (PA006616, CUSBIO), SRC‐3 (ER65603, HUABO), Col1α (BS1530, Bioworld), SP1 (A19649, ABclonal), and GAPDH (FD0063, FDbio). Secondary antibodies included goat anti‐rabbit IgG‐HRP (FDR007, FDbio) and goat anti‐mouse IgG‐HRP (FDM007, FDbio). Proteins were visualized using ECL reagents and detected on an automated chemiluminescence image analysis system (Tanon, China). Band intensities were quantified using ImageJ.

### 2.13 Co‐immunoprecipitation (Co‐IP)

Co‐IP assays were performed in KGN cells following established protocols [26]. Antibodies used for immunoprecipitation included p300 (ab275378, Abcam), BRD4 (83375S, CST), SP1 (A19649, ABclonal), or an isotype‐matched immunoglobulin (Ig) control. Protein A/G Magnetic Beads (PB101, Vazyme, China) were used to pulldown. Immunoprecipitants were washed and analyzed by western blotting.

### 2.14 Reverse Transcription PCR (RT‐PCR)

Total RNA was extracted from ovarian tissues using the Tissue Total RNA Isolation Kit (RC112‐01, Vazyme, China). cDNA was synthesized from equal amounts of RNA using the HiScript RT SuperMix kit (R123‐01, Vazyme, China). PCR amplification was conducted with the following cycling conditions: 95°C for 5 min (initial denaturation), followed by 35 cycles of 95°C for 30 s, 60°C for 30 s, and 72°C for 30 s, with a final extension at 72°C for 5 min. The primers used were: *Ar*: Forward 5′‐TCTGGTTGTCACTACGGAGC‐3′, Reverse 5′‐TGCAATCATTTCTGCTGGCAC‐3′; *Actb* (internal control): Forward 5′‐TTCCTTCCTGGGTATGGAAT‐3′, Reverse 5′‐GAGGAGCAATGATCTTGATC‐3′. For endpoint RT‐PCR, products were resolved on a 2% agarose gel and visualized under UV light.

### 2.15 Chromatin Immunoprecipitation (ChIP)

ChIP assays were performed in ovaries and KGN cells using a commercial kit (P2197M, Beyotime, China) as previously described [26]. Antibodies against SP1 (A19649, ABclonal), p300 (ab275378, Abcam), BRD4 (83375S, CST), H3K18ac (13998T, CST), and H3K27ac (8173T, CST) were used for immunoprecipitation. Input and ChIP‐enriched DNA samples were analyzed by PCR using primers listed in Table S1, which target predicted transcription factor binding motifs within the *Ar/AR* promoter: mouse (*‐46/GGGGGCGGGACCG*) and human (*‐96/GGGGAGGGG*), identified via JASPAR. PCR products were resolved by agarose gel electrophoresis and analyzed using ImageJ.

### 2.16 Statistical Analysis

Data analysis and visualization were performed using GraphPad Prism 9. Normality of the data distribution was assessed using the Shapiro–Wilk test, and homogeneity of variances was tested with Levene's test. For group comparisons, Student's t‐test, one‐way or two‐way ANOVA, followed by Tukey's post‐hoc test, were used as indicated. Data are presented as means ± SEM or as box‐and‐whisker plots. A *p*‐value < 0.05 was considered statistically significant.

## 3 Results

### 3.1 PCOS Ovary Exhibits Abnormal AR Activation, p300 Upregulation, and Histone Acetylation

**FIGURE 1:**
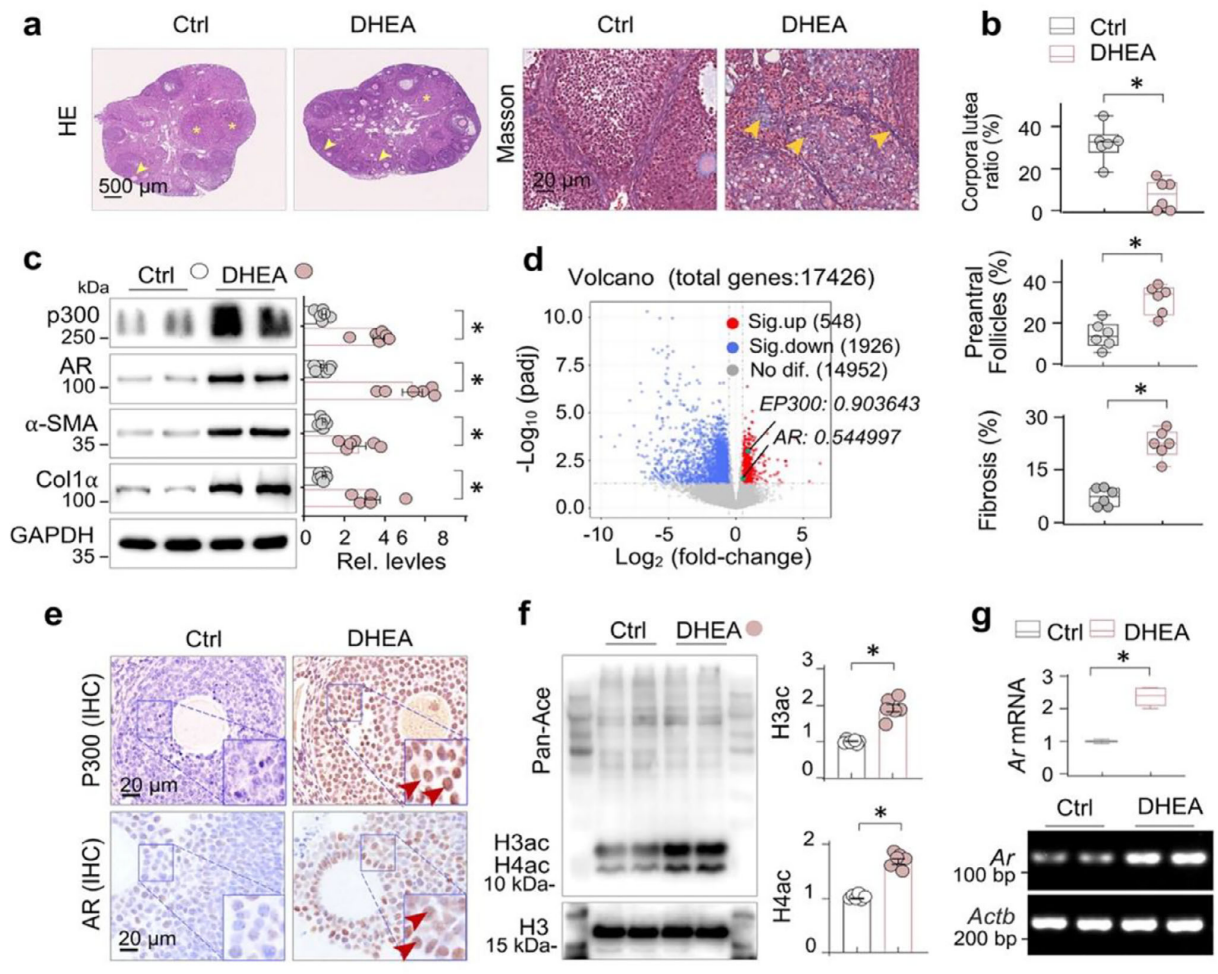
PCOS ovary exhibits abnormal AR activation, p300 upregulation and histone acetylation. Three‐week‐old female C57BL/6J mice were subcutaneously implanted with silicone tubes (1 cm) containing dehydroepiandrosterone (DHEA) for 35 days to establish a PCOS model, the equal‐length empty silicone tubes served as the control (Ctrl) (ten mice in each group). (a) Representative photomicrographs of ovarian sections (Hematoxylin‐eosin and Masson trichrome staining) from Ctrl and DHEA‐treated mice. Corpora lutea were indicated by asterisk, and preantral follicles and the collagen depositions were indicated by arrows. (b) Quantitation of (a). Data were presented as Box‐and‐whisker plots with data points and as means ± SEM, n = 6. *p < 0.05, Student's t‐test. (c) Western blotting. Ovarian tissue homogenates from Ctrl and DHEA‐treated mice were assayed for p300, AR, α‐SMA and Collagen I (Col1α). Two representative samples from each group were shown. GAPDH served as loading control. Quantifications were shown on the right side. Data were presented as means ± SEM, n = 6. *p < 0.05, Student's t‐test. (d) Volcano plot of gene expression profile from the database [GEO Dataset Accession Number: GSE293353, GSE277906, GSE155489, GSE138518], comprising RNA‐seq data ovarian granulosa cells of 15 women diagnosed with PCOS and 15 healthy control women. The number and position of genes statistic‐significantly decreased (1926, blue), no difference (14952, gray) or increased (548, red) including EP300 (Log2 (flod‐change) = 0.903643) and AR (Log2(fold‐change) = 0.544997), green point was marked. (e) Representative photomicrographs of ovarian sections from Ctrl and DHEA‐treated mice stained for p300 and AR by immunohistochemistry (IHC) staining. Positively‐stained granulosa cells were indicated by arrows. (f) Western blotting. Ovarian tissue homogenates were examined for Pan‐Acetyl‐Lysine (Pan‐Ac). Two representative samples from each group were shown. H3 served as loading control. Quantifications of histone 3 and histone acetylation were on the right side. Data were presented as means ± SEM, n = 6. *p < 0.05, Student's *t*‐test. (g) RT‐PCR of ovarian tissues from Ctrl and DHEA‐treated mice (n = 6) for Ar mRNA. Beta‐actin gene Actb served as internal control. Two samples from each group were shown. Quantifications were on the upper side. Data were presented as means ± SEM. *p < 0.05, Student's *t*‐test.

As a first step to investigate the role of p300 in the development of PCOS mice, we adapted a PCOS mouse model. This model was established by subcutaneous implantation of a slow‐release silicone tube containing DHEA. Histological examination revealed a reduced number of corpora lutea, an increased number of preantral follicles, and substantial fibrotic collagen deposition in the ovaries of PCOS mice (Figure 1a,b). Moreover, the expression levels of p300, AR, and the fibrotic factor α‐SMA and type I collagen (Col1α) were all upregulated in PCOS mouse ovaries (Figure 1c). Consistently, a differential RNA‐seq analysis of ovarian granulosa cells from 15 women with PCOS and 15 healthy controls, acquired from the GEO database, further demonstrated a significant increase in *EP300* (Log_2_FC = 0.903643) and *AR* (Log_2_FC = 0.544997) expression in the PCOS women (Figure 1d). Immunohistochemical staining revealed increased expression of both p300 and AR in the granulosa cells (GCs) of PCOS ovaries (Figure 1e), accompanied by increased acetylation of histone 3 and histone 4 (Figure 1f). Moreover, agarose gel electrophoresis and quantitative RT‐PCR analysis confirmed a significant increase in *Ar* mRNA levels (Figure 1g), indicating that p300 upregulates AR at the transcriptional level. These results suggest that p300 may play a critical role in driving ovarian fibrosis in the context of PCOS.

### 3.2 p300‐driven H3K18ac and H3K27ac Upregulate AR Transcription in Granulosa Cells of Mouse PCOS Ovaries

**FIGURE 2:**
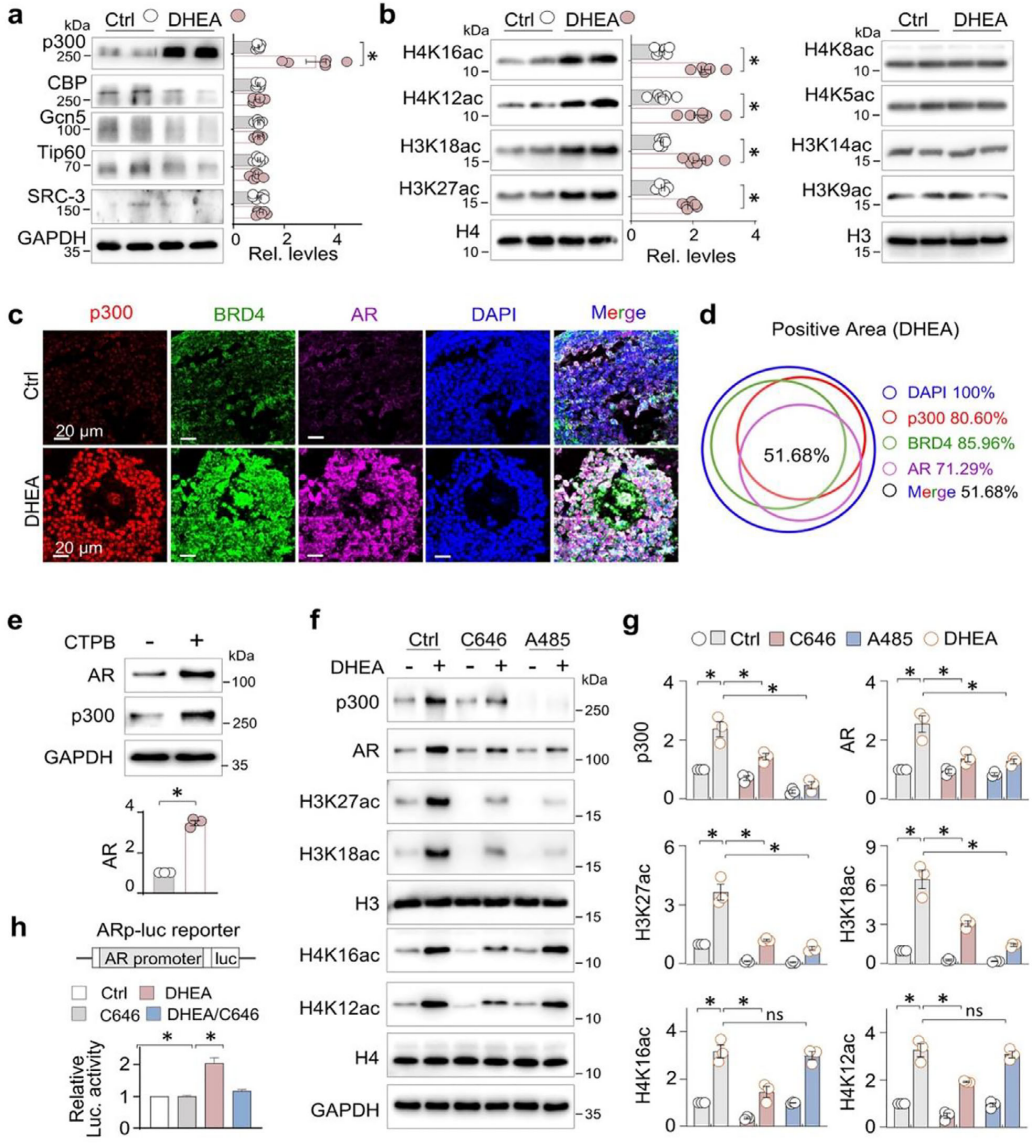
p300‐driven H3K18ac and H3K27ac upregulates AR transcription in granulosa cells of mouse PCOS ovaries. (a) Western blotting. Ovarian tissue homogenates from Ctrl and DHEA‐treated mice were examined for p300, CBP, Gcn5, Tip60 and SRC‐3. GAPDH served as loading control. Two representative samples from each group were shown. Quantifications were shown on the right side. Data were presented as means ± SEM, n = 6. *p < 0.05, Student's t‐test. (b) Western blotting. Ovarian tissue homogenates were assayed for H4 acetylation (H4K16ac, H4K12ac, H3K18ac, H3K27ac) and H4 (the left panel), and H3 acetylation (H4K8ac, H4K5ac, H3K14ac, H3K9ac) and H3 (the right panel). Quantifications were shown. Data were presented as means ± SEM, n = 6. *p < 0.05, Student's t‐test. (c) Representative photomicrographs of ovarian sections from Ctrl and DHEA‐treated mice stained for p300 (red), BRD4 (green) and AR (magenta) by multiplex immunofluorescence (mIF) staining. Cell nuclei were stained with DAPI. (d) Quantifications of (c). The percentage of each fluorescent positive area relative to the DAPI area. (e) Primary granulosa cells (GCs) were treated with CTPB (5 µm, 24 h). The cell lysates were assayed for p300 and AR. Quantifications were shown on the lower side. Data were presented as means ± SEM, n = 3, *p < 0.05, Student's *t*‐test. (f) Primary granulosa cells (GCs) were treated with DHEA (25 µm, 48 h) followed by C646 (10 µm, 24 h) or A‐485 (1 µm, 24 h) treatment, and then cell lysates were assayed for p300, AR, H3K18ac, H3K27ac, H4K16ac, H4K12ac, H3, H4, and GAPDH. (g) Quantification of (e). Data were presented as means ± SEM, n = 4, *P < 0.05, two‐way ANOVA followed by Tukey's post‐hoc test. (h) Luciferase assay. HEK293T cells were transfected with an AR promoter‐luciferase reporter AR‐luc plus a renilla luciferase reporter, and then treated with DHEA (25 µm, 48 h) with or without C646 (10 µm, 24 h). Cell lysates were assayed for luciferase activities. The relative luciferase activities of fold changes were presented. Data were presented as means ± SEM, n = 4, *P < 0.05, one‐way ANOVA followed by Tukey's post‐hoc test.

We further found that p300, among the histone acetyltransferase (HAT) family members, including CBP, Gcn5, Tip60, and SRC‐3, was preferentially upregulated in the ovaries of PCOS mice (Figure 2a). This upregulation coincided with a significant increase in acetylation at lysine residues 18 and 27 on histone 3 (H3K18ac/H3K27ac) and at lysine residues 12 and 16 on histone 4 (H4K12ac/H4K16ac), whereas acetylation at H4K5, H4K8, H3K9, and H3K14 remained unchanged (Figure 2b). Notably, BRD4 – a bromodomain‐containing protein and acetyl‐reader known to regulate AR transcription – was found to co‐localized with both p300 and AR within the GC of PCOS ovaries (Figure 2c), and the co‐localized positive area accounted for 51.68% of the DAPI area (Figure 2d), suggesting a potential collaborative role in modulating AR transcriptional.

To test whether elevated p300 directly drives AR activation, we modulated p300 activity in vitro. Activation of p300 by treating GCs with the agonist CTPB significantly increased AR expression (Figure 2e). Furthermore, DHEA‐treated GCs using increasing concentrations of A‐485 (0.1, 0.5, 1, 5, and 10 µm), a potent and specific p300 inhibitor, showed significant downregulation of H3K18ac and H3K27ac in a dose‐dependent manner, while H4K12ac and H4K16ac levels remained largely unchanged. Notably, A‐485 also reversed DHEA‐induced AR upregulation (Figure S1). In addition, 1 µM A‐485 achieved more potent and selective inhibition of H3K18ac, H3K27ac, and AR upregulation than 10 µm C646 in DHEA‐treated granulosa cells (Figure 2f,g). These findings indicate that p300‐mediated acetylation of H3K18/H3K27 is primarily responsible for AR activation in this model. We further assessed AR promoter activity using a luciferase reporter system (AR‐luc). C646 treatment significantly suppressed DHEA‐stimulated AR‐luc transactivation (Figure 2h). These converging results demonstrate that p300 promotes AR transcription in PCOS granulosa cells by increasing acetylation at H3K18 and H3K27, establishing p300 as a key upstream regulator of AR signaling in PCOS.

### 3.3 p300 knockout in Granulosa Cells Resists Ovarian AR Activation and Fibrosis in PCOS Mice

**FIGURE 3:**
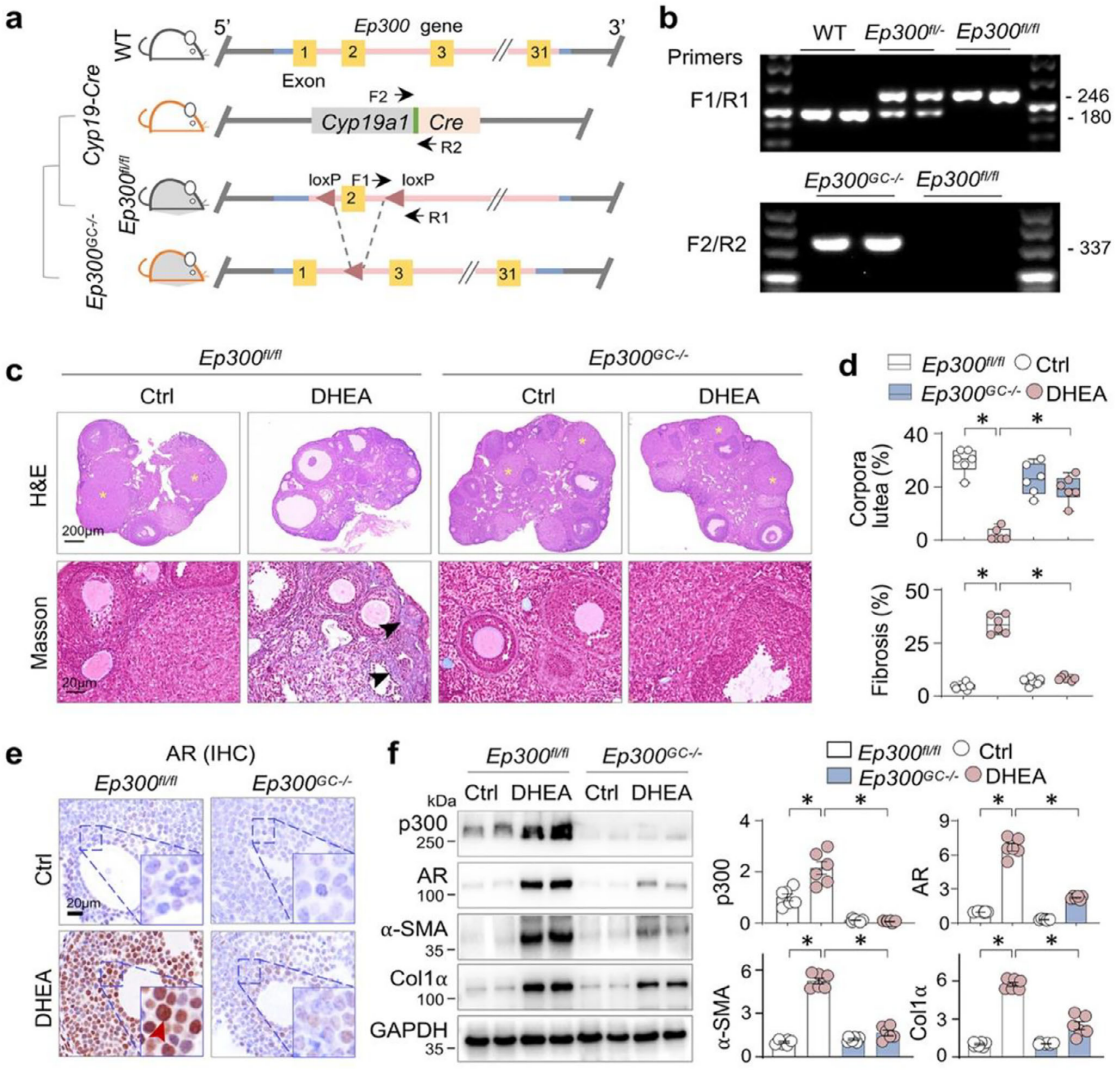
p300 knockout in granulosa cells resists ovarian AR activation and fibrosis in PCOS mice. (a) Generation of conditional *Ep300* knockout mice (*Ep300^GC^* ^‐/−^) by crossing *Cyp19a1*‐*Cre* with *Ep300* ^fl/fl^ mice. *Ep300* locus in wild‐type mice were represented by boxes (exons 1–31). The positions of *Cyp19a1*‐*Cre* genotyping PCR primers F2 and R2 (arrows), and the loxP sites (triangles) in *Ep300* ^fl/fl^ mice and the genotyping PCR primers F1 and R1 (arrows) were depicted. (b) Agarose gel representation of genotyping of WT, *Ep300* ^fl/−^, *Ep300* ^fl/fl^ and *Ep300^GC^* ^‐/−^ mice. (c) Representative photomicrographs of ovarian sections (Hematoxylin‐eosin and Masson trichrome staining) from *Ep300* ^fl/fl^ and *Ep300^GC^* ^‐/−^ mice treated with subcutaneous implantation of silicone implants (1 cm) for sustained release of dehydroepiandrosterone (DHEA) for 35 days (n = 6). (d) Quantification of (c). The corpora lutea and collagen deposition were indicated by asterisk or arrows, respectively. Data were presented as Box‐and‐whisker plots with data points and as means ± SEM; n = 6, *p < 0.05, one‐way ANOVA followed by Tukey's post‐hoc test. (e) Representative photomicrographs of ovarian sections from *Ep300* ^fl/fl^ and *Ep300^GC^* ^‐/−^ mice treated with DHEA stained for AR by immunohistochemistry (IHC) staining. Positively‐stained granulosa cells were indicated by arrows. (f) Western blotting. The ovarian tissue homogenates were assayed for p300, AR, α‐SMA, Collagen I (Col1α) and GAPDH. Two randomly‐selected samples from each group were shown. Quantifications were shown on the right side. Data were presented as means ± SEM; n = 6, *p < 0.05, one‐way ANOVA followed by Tukey's post‐hoc test.

To determine the functional impact of p300 dysregulation on AR activation and ovarian fibrosis in PCOS, we generated a strain of GC‐specific *Ep300* knockout mice (*Ep300* ^fl/fl^; *Cyp19a1*‐*Cre* (*Ep300^GC^* ^‐/−^)) (Figure 3a) and verified the mice by benotyping (Figure 3b). *Ep300^GC^* ^−/−^ mice had normal ovarian morphology. However, compared to *Ep300* ^fl/fl^ PCOS mice, *Ep300^GC^* ^‐/−^ PCOS mice exhibited a higher number of corpora lutea, reduced fibrotic collagen deposition (Figure 3c,d). Furthermore, the area of AR‐positive staining in granulosa cells was significantly lower in *Ep300^GC^* ^‐/−^ PCOS mice than in *Ep300* ^fl/fl^ PCOS mice (Figure 3e). Western blot analysis confirmed efficient p300 knockout and demonstrated that AR, α‐SMA, and Col1α were all reduced in *Ep300^GC^* ^−/−^ PCOS ovaries (Figure 3f). These data suggest that p300 plays a functional role in promoting AR activation and fibrosis in PCOS ovaries.

### 3.4 Pharmacological Inhibition of p300 Alleviates Abnormal AR Activation and Ovarian Fibrosis in PCOS Mice

**FIGURE 4:**
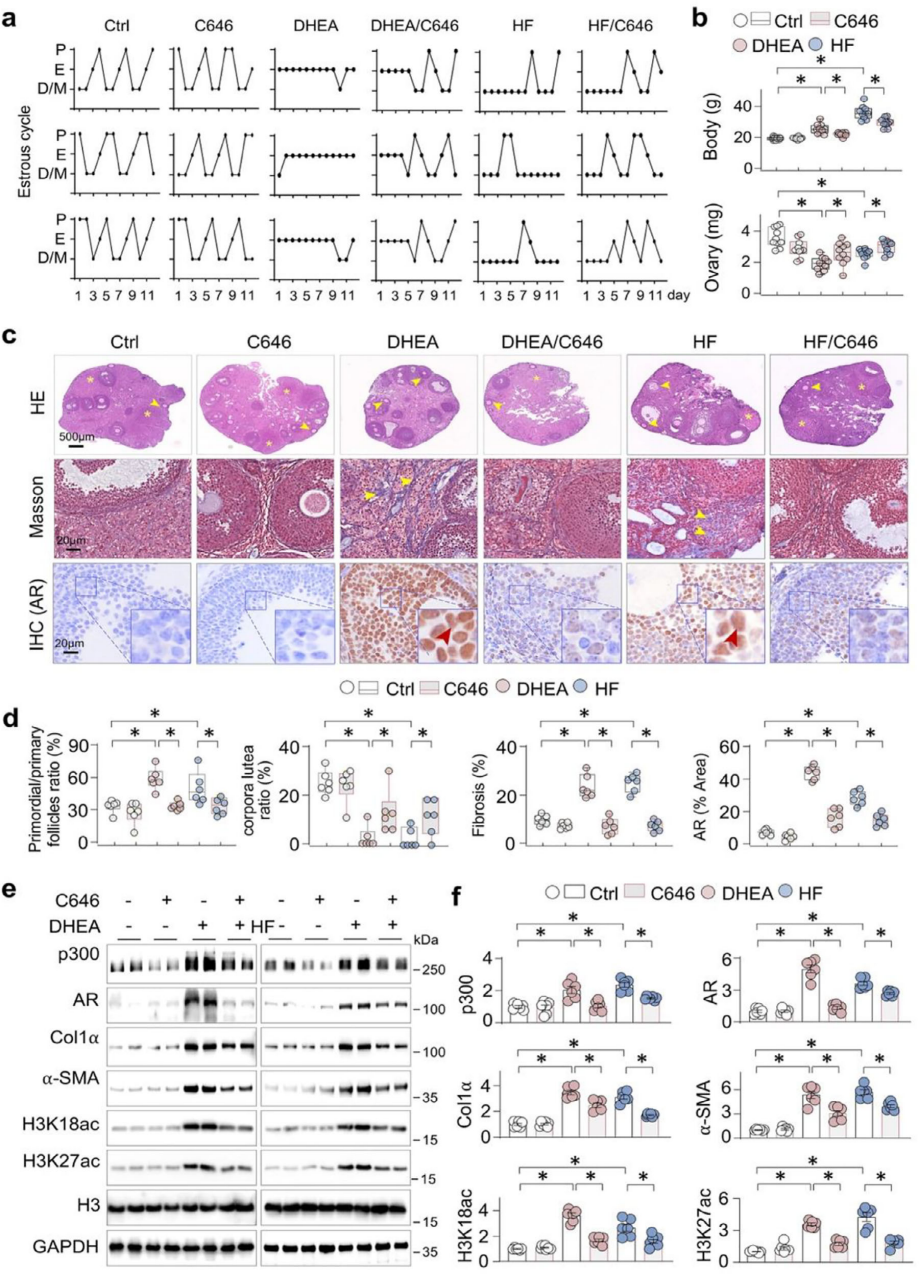
Pharmacological inhibition of p300 alleviates abnormal AR activation and ovarian fibrosis in PCOS mice. Two PCOS models were established in three‐week‐old female C57BL/6J mice by either subcutaneous implantation of dehydroepiandrosterone (DHEA)‐containing silicone tubes (1 cm) for 35 days or by feeding a high‐fat diet (HF, 60% fat) for 3 months. Ten days prior to the completion of the modeling period, mice were injected intraperitoneally with the p300 inhibitor C646 (10 mg/kg) (ten mice in each group). (a) Representative estrous cyclicity of three mice each group during 12 consecutive days. P: proestrus, E: estrus, D/M: diestrus phase/metestrus. (b) Body weight (g) (the upper side) and ovary weight (mg) (the lower side). Data were presented as means ± SEM; n = 10, *p < 0.05, one‐way ANOVA followed by Tukey's post‐hoc test. (c) Representative photomicrographs of ovarian sections stained by Hematoxylin‐eosin (the upper pictures), Masson trichrome (the middle pictures) or immunohistochemistry of AR (the lower pictures). The corpora lutea were indicated by asterisk, and primordial/primary follicles, collagen deposition and positively‐stained granulosa cells were indicated by arrows. (d) Quantifications of primordial/primary follicles ratio and corpora lutea ratio, ovarian fibrosis staining and AR positively‐stained granulosa cells in (c). Data were presented as Box‐and‐whisker plots with data points. Data were presented as means ± SEM; n = 6, *p < 0.05, one‐way ANOVA followed by Tukey's post‐hoc test. (e) Western blotting. The ovarian tissue homogenates were assayed for p300, AR, α‐SMA, Collagen I (Col1α), H3K18ac, H3K27ac, H3 and GAPDH. Two randomly‐selected samples from each group were shown. (f) Quantifications of (e). Data were presented as means ± SEM. n = 6, *p < 0.05, one‐way ANOVA followed by Tukey's post‐hoc test.

To further explore the role of p300 in AR activation and ovarian fibrosis, we constructed two PCOS mouse models with DHEA or a high‐fat diet (HF), and treated PCOS mice with C646, a p300‐selective inhibitor. C646 administration significantly restored estrous cycle regularity (Figure 4a), reduced mouse body weight, and increased ovary weight (Figure 4b). In addition, C646 treatment effectively increased the number of corpora lutea, decreased the number of primordial or primary follicles, and reduced fibrotic collagen deposition in PCOS mice (Figure 4c,d), indicating improved ovarian function. Consistently, C646 treatment markedly normalized the expression of AR, p300, fibrotic factors Col1α and α‐SMA, and acetylation levels of H3K18 and H3K27 in PCOS ovaries (Figure 4e,f). These results suggest that selective inhibition of p300 alleviates ovarian fibrosis and dysfunction in PCOS, potentially through the suppression of AR signaling.

### 3.5 AR Transactivation Is Linked to Increased Chromatin Accessibility and SP1 Footprint Dynamics

**FIGURE 5:**
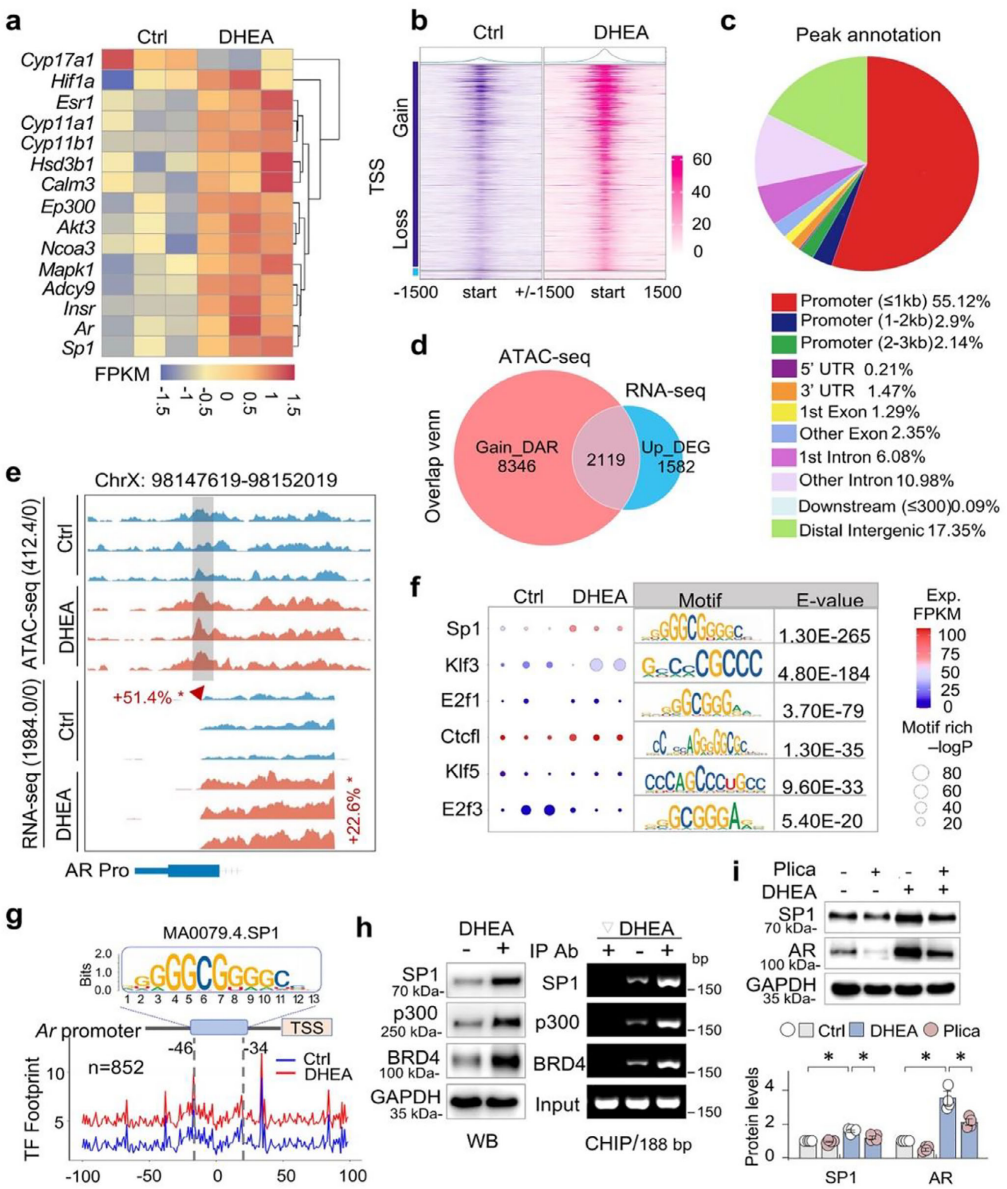
AR transactivation is linked to increased chromatin accessibility and SP1 footprint dynamics. Three‐week‐old female C57BL/6J mice were subcutaneously implanted with silicone tubes (1 cm) containing dehydroepiandrosterone (DHEA) for 35 days to establish a PCOS model, the equal‐length empty silicone tubes served as the control (Ctrl). Ovarian tissues were collected for ATAC‐seq and RNA‐seq analysis (n = 3 in each group). (a) Heatmap of normalized expression (FPKM) for 15 key genes associated with estrogen signaling, cortisol synthesis and secretion, HIF‐1α signaling, and oocyte meiosis in Ctrl and DHEA‐treated mice by RNA‐seq analysis. (b) Open chromatin regions surrounding gene transcription start sites (TSSs) in ovarian tissues from Ctrl and DHEA‐treated mice by ATAC‐seq analysis. (c) Pie chart depicting the peaks annotation identified in the ATAC‐seq analysis. The chart illustrates the distribution of accessible chromatin regions across various functional elements in the genome. (d) Venn diagram illustrating the overlap between gene with increased peaks (ATAC‐seq, *p < 0.05*) and genes with increased expression (RNA‐seq, |log2FC| > 1, *p < 0.05*) in Ctrl vs. DHEA‐treated mice. (e) Peak plot showing the ATAC‐seq peaks (normalized reads) and RNA‐seq mRNA expression (FPKM) at the *Ar* locus (ChrX: 98147619–98152019) in Ctrl (blue) vs. DHEA‐treated (red) mice. The peaks exhibiting a specific increase upon DHEA treatment are indicated by gray boxes and red asterisk. (f) A heatmap displays the top six transcription factors (TFs) binding to the *Ar* promoter region in the ATAC‐seq analysis, along with their mRNA expression identified by RNA‐seq analysis, and the predicted TF motifs were shown on the right. (g) Aggregated footprint plot of SP1. The aggregated plot depicts the predicted SP1 binding motifs across 852 total possibilities, comparing the Ctrl and DHEA conditions. The dashed lines indicate the boundaries of the SP1 motif at the *Ar* promoter. The plot visualizes the change in chromatin accessibility and transcription factor (TF) footprints across the different treatments. (h) Western blotting (the left side) and ChIP assay (the right side). Western blotting assay, the ovarian tissue homogenates from Ctrl and DHEA‐treated mice were assayed for SP1, p300, BRD4 and GAPDH. ChIP assay, the ovarian tissue homogenates were immunoprecipitated with isoform‐matched immunoglobulin or antibodies to SP1, p300 or BRD4, respectively, and then the genomic DNA (Input) and the antibody‐bound DNAs were PCR‐amplified with primers covering the SP1 motif on *Ar* promoter. The PCR products were analyzed on 1.5% agarose gels. (i) Western blotting. KGN cells were treated with DHEA (25 µm, 48 h) with or without Plicamycin (Plica, 100 nm, 24 h), and then cell lysates were assayed for SP1, AR and GAPDH. The quantification graph is shown below. Data were presented as means ± SEM. n = 4, *p < 0.05, one‐way ANOVA followed by Tukey's post‐hoc test.

To investigate how p300 enhances AR expression, we performed RNA‐seq and ATAC‐seq on ovaries from DHEA‐treated PCOS mice. RNA‐seq revealed upregulation of both *Ep300* and *Ar*, among 15 genes implicated in estrogen signaling and PCOS pathogenesis (Figure 5a). ATAC‐seq showed a global increase in chromatin accessibility (Figure 5b), with most gains occurring near promoter proximal regions (≤ 1 kb of TSS, 55.12%) (Figure 5c). As expected, *Ar* was amongst 2119 genes with both elevated mRNA and increased chromatin accessibility (Figure 5d,e). At the *Ar* locus, chromatin accessibility increased by 51.4% near the transcriptional start site (TSS), correlating with a 22.6% rise in transcription levels (Figure 5e).

Further motif analysis of the *Ar* promoter identified several transcription factor (TF) binding sites, including *Klf3*, *E2f1*, *Ctcf1*, *Klf5*, and *E2f3*, with *Sp1* showing the highest enrichment (E‐value = 1.30E‐265, Figure 5f). SP1 expression was also elevated in DHEA‐treated ovaries (FPKM values, 69.714 vs. 55.524, Figure 5f). The identification of an SP1 binding motif (‐46/gggggcgggacc) on the *Ar* promoter suggests transcriptional regulation of AR by SP1. This was corroborated by transcription factor footprinting analysis; increased Tn5 transposase insertion at the SP1 binding motif following DHEA treatment confirmed active SP1 occupancy at the *Ar* promoter (Figure 5g). Western blot and ChIP assays confirmed increased expression and inducible promoter binding of SP1, p300, and BRD4 to the *Ar* promoter in PCOS ovaries (Figure 5h). Moreover, treatment of DHEA‐stimulated granulosa cells with the SP1‐selective inhibitor Plicamycin significantly reduced AR levels (Figure 5i). These findings suggest that SP1 acts as a key cofactor in p300‐driven AR transcriptional activation in PCOS.

### 3.6 P300 forms a Transcriptional Complexes With SP1 and BRD4 to Drive AR Activation

**FIGURE 6:**
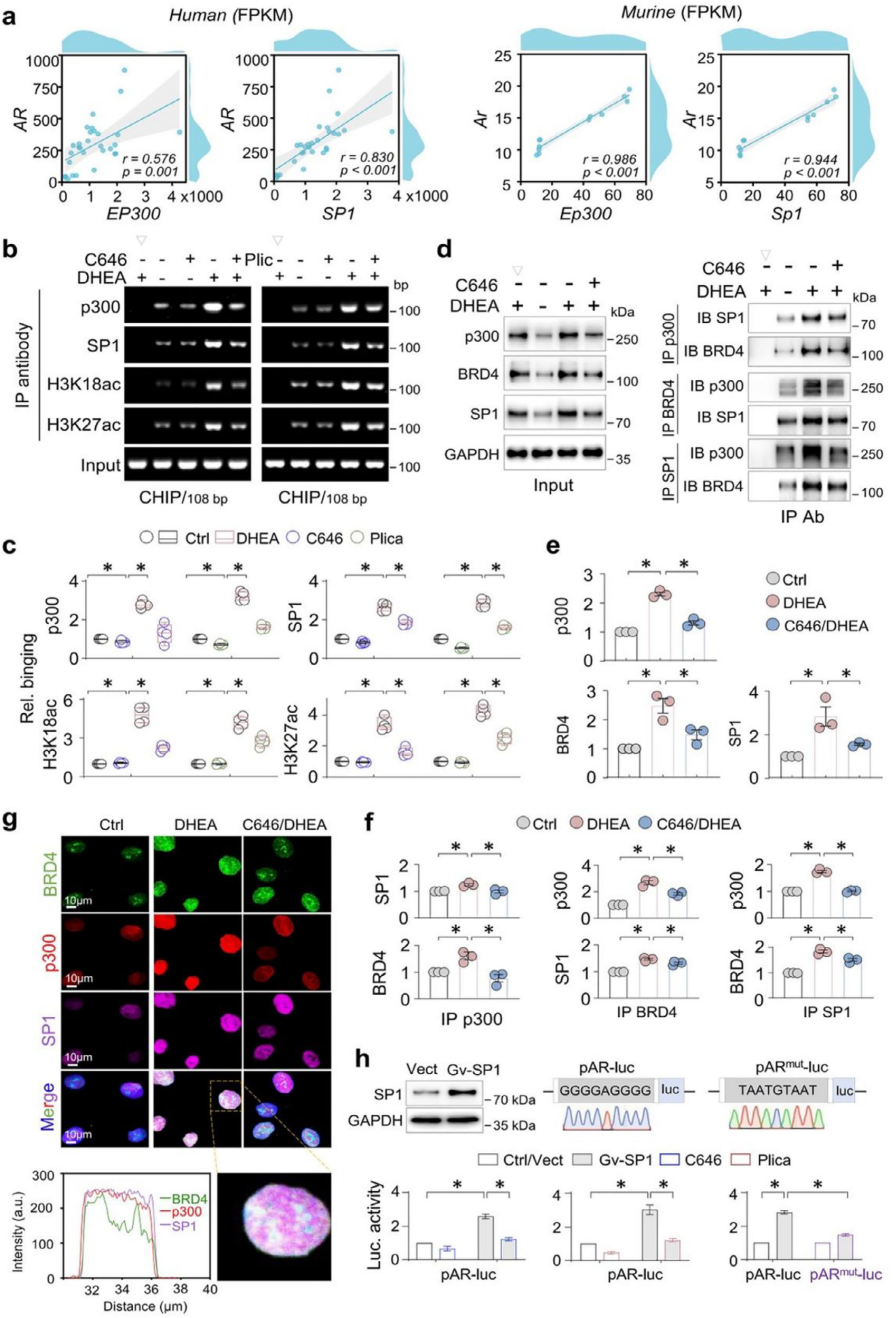
p300 forms a transcriptional complexes with SP1 and BRD4 to drive AR activation. (a) Scatter plots showing *EP300*‐*AR* (*Ep300*‐*Ar*) and *SP1*‐*AR* (*Sp1*‐*Ar*) expression correlations in human ovarian granulosa cells (the left panel) and murine ovaries (the right panel). (b) ChIP assay. KGN cells were treated with DHEA (25 µm, 48 h) in presence or absence of C646 (10 µm, 24 h) or Plicamycin (Pli, 100 nm, 24 h), respectively, and then cell lysates were immunoprecipitated with isoform‐matched immunoglobulin or antibodies to p300, SP1, H3K18ac, or H3K27ac, respectively, and then the genomic DNA (Input) and the antibody‐bound DNAs were PCR‐amplified with primers covering the SP1 motif on *AR* promoter. The PCR products were analyzed on 1.5% agarose gels. (c) Quantification of (b). Data were presented as Box‐and‐whisker plots with data points and as means ± SEM; n = 4, *P < 0.05, one‐way ANOVA followed by Tukey's post‐hoc test. (d) Co‐IP assay. KGN cells were treated with DHEA (25 µm, 48 h) with or without C646 (10 µm, 24 h), and then cell lysates were immunoprecipitated with isoform‐matched immunoglobulin (Ig) or antibody (IP Ab) to p300, BRD4, or SP1, and then immunoprecipitants were assessed for p300, BRD4, or SP1 by western blotting reciprocally (the upper panel). The non‐IP lysates were assayed for p300, BRD4, SP1, and GAPDH as input controls. (e) Quantification of the Input bands in (d). Data were presented as means ± SEM; n = 3, *P < 0.05, one‐way ANOVA followed by Tukey's post‐hoc test. (f) Quantification of the IP bands in (d). Data were presented as means ± SEM; n = 3, *P < 0.05, one‐way ANOVA followed by Tukey's post‐hoc test. (g) KGN cells were treated with DHEA (25 µm, 48 h) with or without C646 (10 µm, 24 h), and then were stained for p300 (red), BRD4 (green) and SP1 (magenta) by multiplex immunofluorescence (mIF) staining. The colocalization analysis peak plot is shown below. (h) HEK293T cells were transfected with an *AR* promoter‐luciferase reporter AR‐luc (GGGGAGGGG) or a mutant *AR* promoter reporter ARmut‐luc (TAATGTAAT) plus a renilla luciferase reporter for 24 h, in presence or absence of a plasmid overexpressing SP1 (Gv‐SP1). At the same time, cells were treated with or without C646 (10 µm, 24 h) or Plicamycin (Plica, 100 nm, 24 h), respectively. Then cell lysates were assayed for SP1 and GAPDH (Western blotting) or luciferase activities. The relative luciferase activities of fold changes were presented. Data were presented as means ± SEM; n = 4, *P < 0.05, two‐way ANOVA followed by Tukey's post‐hoc test.

To clarify how elevated p300 and SP1 contribute to AR activation in PCOS, we analyzed RNA‐seq data from human ovarian granulosa cells and mouse ovaries (GEO database). Spearman correlation analysis revealed strong positive correlations between *EP300*/*Ep300* and *AR*/*Ar* expression (human, r = 0.576, p = 0.001; Murine, r = 0.986, p < 0.001), as well as between *SP1*/*Sp1* and *AR*/*Ar* (human, r = 0.830, p < 0.001; murine, r = 0.944, p < 0.001) (Figure 6a). ChIP assays in KGN cells confirmed inducible binding of SP1 and p300 to the SP1 motif‐containing *AR* promoter, marked by active histone modifications H3K18ac and H3K27ac. This binding was suppressed by both the SP1 inhibitor Plicamycin and the p300‐selective inhibitor C646 (Figure 6b,c), supporting a direct regulatory role of SP1 in AR transcription.

Given that p300 operates through a transcriptional complex that guides it to specific gene promoters [39], we investigated its interactions in PCOS ovaries by Co‐IP and found inducible associations between p300, SP1, and BRD4 in PCOS ovaries (Figure 6d–f). Immunofluorescence co‐localization in DHEA‐treated KGN cells further validated the aberrant nuclear co‐expression of these factors, which was reversed by C646 (Figure 6g). To functionally assess SP1's role, we overexpressed SP1 via Gv‐SP1 plasmid transfection in HEK293T cells. This led to increased AR‐luceferase reporter activity, which was reversed by Plicamycin and C646 (Figure 6h, the left and middle panels). We also made a mutant version (AR^mut^‐luc) of the wild‐type reporter (AR^wt^‐luc) in which the SP1 binding motif (‐116/ GGGGAGGGG) was replaced by a no‐binding sequences (TAATGTAAT). SP1 overexpression significantly activated AR^wt^‐luc, while AR^mut^‐luc showed minimal response (Figure 6h, the right panel). Collectively, these findings demonstrate that p300 promotes AR transcriptional activation in PCOS through complex formation with SP1 and BRD4 at the AR promoter.

### 3.7 AR Activation Undermines the Anti‐fibrosis Effects of p300 Inhibition in Ovaries of PCOS Ovaries

**FIGURE 7:**
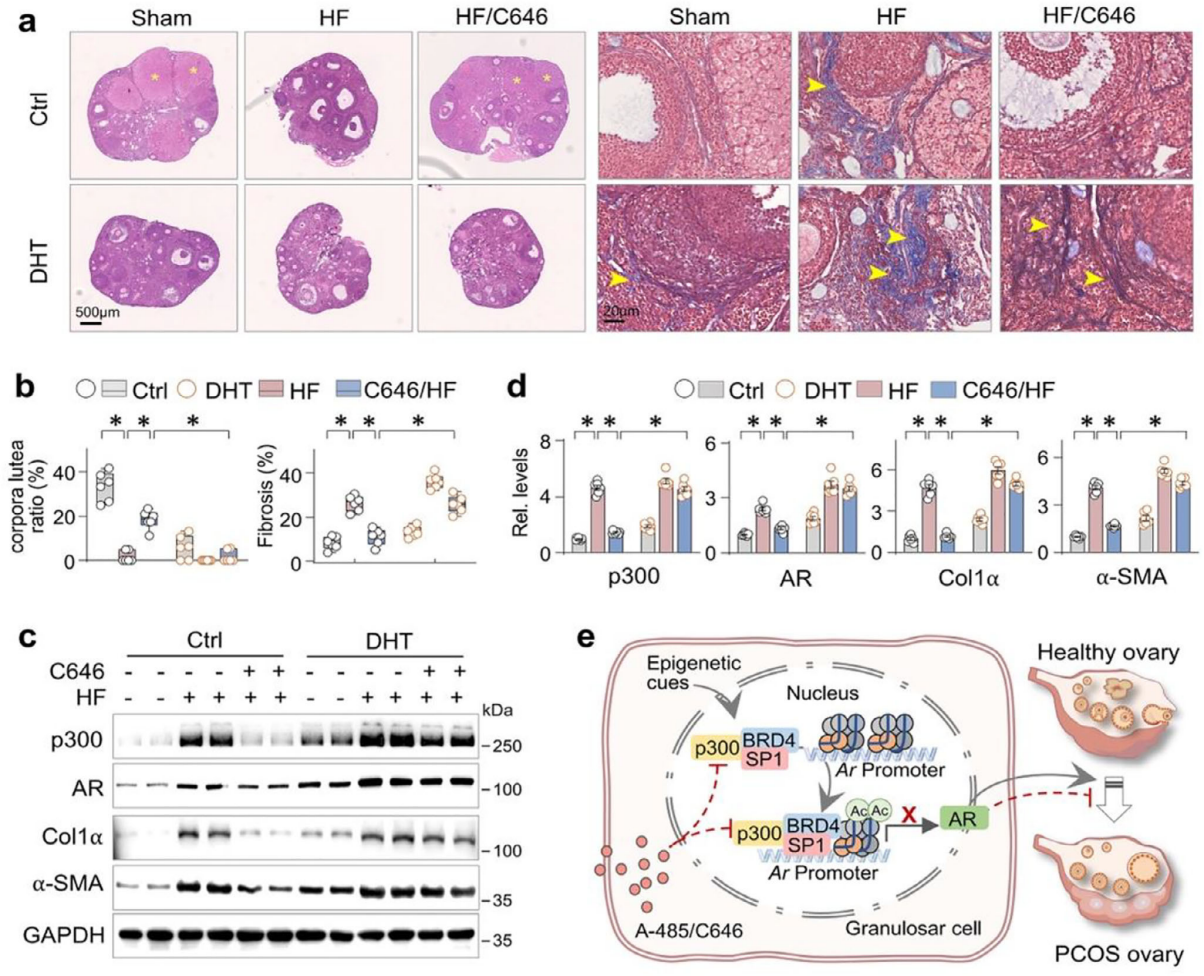
AR activation undermines the anti‐fibrosis effects of p300 inhibition in ovaries of PCOS ovaries. Three‐week‐old female C57BL/6J mice were accepted high‐fat diet (HF, 60% fat) for 3 months, and mice receiving Control vehicle (Ctrl) or DHT (1.66 mg/kg, 10 days) were grouped into Control (Ctrl), C646, high‐fat diet (HF), and C646‐treated HF mice (ten mice in each group). (a) Representative microphotographs of ovarian sections stained by Hematoxylin‐eosin (the left panel) and Masson trichrome (the right panel). The corpora lutea and collagen deposition were indicated by asterisk or arrows, respectively. (b) Quantitation of corpora lutea ratio and fibrosis staining of (a). Data were presented as Box‐and‐whisker plots with data points. Data were presented as means ± SEM; n = 6, *p < 0.05, two‐way ANOVA followed by Tukey's post‐hoc test. (c) Western blotting. The ovarian tissues from the experimental mice in (a) were assayed for p300, AR, Collagen I (Col1α), α‐SMA, and GAPDH. Two randomly‐selected samples from each group were shown. (d) Quantification of (c). Data were presented as means ± SEM. n = 6, *p < 0.05, two‐way ANOVA followed by Tukey's post‐hoc test. (e) A schematic of sequential p300 induction of histone 3 acetylation, formation of p300/BRD4/SP1 complex and AR transactivation, leading to PCOS. P300 inhibition with A‐485/C646 blocks the process.

Since C646 broadly affects p300‐regulated gene expression, we aimed to determine whether its protective effect on ovarian fibrosis depends specifically on AR suppression. To test this, we compared the anti‐fibrotic and pro‐ovulatory effects of C646 in mice with or without exposure to the AR agonist DHT. Mice were divided into three groups: Sham, high‐fat diet (HF), and HF + C646, with or without DHT co‐treatment. In HF mice, C646 significantly increased corpus luteum formation and reduced ovarian fibrosis, as shown by H&E and Masson staining (Figure 7a,b). However, these effects were markedly attenuated in DHT‐exposed HF mice (Figure 7a,b). At the molecular levels, C646 reversed the elevated expression of p300, AR, Col1α, and α‐SMA in HF mice, but this suppression was substantially weakened in the presence of DHT (Figure 7c,d). Together, these findings suggest that AR activation plays a central role in mediating ovarian fibrosis and dysfunction in PCOS, and that p300 inhibition alleviates these pathologies primarily through suppression of AR signaling.

## 4 Discussion

This study reveals a pivotal epigenetic regulatory role of the histone acetyltransferase p300 in driving AR activation and ovarian fibrosis in PCOS. We found that p300 specifically acetylates histone 3 at lysine 18 and 27 (H3K18ac and H3K27ac), creating an “open” chromatin configuration at the *A*r promoter, and facilitates AR transcription through the assembly of a transcriptional activation complex with BRD4 and SP1. Conversely, both granulosa cell‐specific ablation of p300 and pharmacological blockage with A‐485 or C646 effectively inhibited AR activation, attenuated ovarian fibrosis, and restored follicle development and ovulation in PCOS mouse models (Figure 7e). Thus, our study reveals an important epigenetic pathway in PCOS, whereby the upregulation of p300 and its activation of AR drive ovarian fibrosis and follicular arrest.

Understanding the upstream regulators, cofactors, and molecular targets that drive epigenetic disorders is essential for uncovering disease mechanisms and exploring intervention strategies [40, 41]. This study identifies p300 as a central epigenetic regulator of AR transcriptional activation in PCOS, acting through selective hyperacetylation of H3K18 and H3K27 at the *Ar* promoter. We show that increased p300 activity in ovarian granulosa cells promotes AR‐driven pathology, including fibrosis and follicular dysfunction. Both genetic deletion and pharmacological inhibition of p300 suppressed AR activation and alleviated PCOS‐like phenotypes in mouse models. Moreover, our findings indicate that p300 forms a transcriptional complex with SP1 and BRD4, maintaining an open chromatin state that sustains aberrant AR expression. Inhibition of SP1 disrupts this complex and suppresses AR activation. Together, these results reveal a disease‐specific epigenetic mechanism in PCOS and highlight the p300–SP1–BRD4 axis as a promising therapeutic target for disrupting pathogenic AR signaling and restoring ovarian function.

Among the diverse families of histone acetyltransferases (HATs), including Gcn5, MYST, TAFII250, and nuclear hormone receptor‐associated HATs, p300 stands out for its exceptional versatility and multifunctionality [42, 43]. Beyond catalyzing classical histone acetylation, p300 also mediates a growing range of acylation modifications, such as lactylation, crotonylation, butyrylation, and propionylation [44]. These capabilities position it as a central epigenetic regulator of gene expression and cellular fate [45]. Although histone acetylation is counteracted by histone deacetylases (HDACs), we found that p300 is selectively upregulated in the PCOS ovary, where it plays a direct and pathogenic role. Specifically, it promotes AR transcriptional activation via histone 3 acetylation at lysines 18 and 27 (H3K18ac and H3K27ac). We showed that genetic deletion of *Ep300* in granulosa cells, as well as pharmacological inhibition with the p300‐specific inhibitor C646, effectively reduced ovarian AR activation and fibrosis in PCOS mouse models. These results point to a disease‐specific epigenetic reprogramming in which p300 is aberrantly unregulated and functionally hijacked to drive AR activation, ovarian fibrosis, and follicular arrest—hallmarks of PCOS. Given p300's broader acrylation activity, further exploration into its non‐acetylation modifications may reveal new biomarkers and therapeutic targets [46]. Overall, this study places p300 at the intersection of epigenetic regulation and endocrine dysfunction, highlighting it as a promising target for next‐generation epigenetic therapies in PCOS.

Histone acetylation is a pivotal epigenetic modification that regulates gene expression by loosening chromatin structure and facilitating the recruitment of transcriptional machinery [47]. Notably, specific acetylation marks exhibit distinct and context‐dependent patterns, with acetylation at H3K9ac, H3K14ac, H3K18ac, H3K27ac, as well as H4K5ac, H4K8ac, H4K12ac, H4K16ac widely recognized as hallmarks of transcriptional activation across various cell types and disease states [47, 48]. In the PCOS mouse ovary, we observed significant upregulation of canonical activation‐associated marks, including H3K27ac, H3K18ac, H4K12ac, and H4K16ac. Intriguingly, while the p300 inhibitor C646 reduced all these marks, the more potent and specific p300 inhibitor A‐485 specifically downregulated H3K18ac and H3K27ac, with minimal effect on H4K12ac and H4K16ac, and concomitantly reversed DHEA‐induced AR upregulation in granulosa cells. These findings indicate that p300‐mediated acetylation of H3K18/H3K27 is primarily responsible for driving AR activation in this model, highlighting the selective nature of epigenetic remodeling in PCOS. Collectively, our results delineate the p300–H3K18/H3K27 acetylation–AR axis as a key pathogenic pathway in PCOS and broaden our understanding of histone modification specificity in reproductive disorders.

## 5 Conclusions

Our study identifies a disease‐specific epigenetic mechanism in PCOS, driven by p300‐mediated H3K18 and H3K27 acetylation at the AR promoter. This process is facilitated by a transcriptional complex involving SP1 and BRD4, promoting aberrant AR activation and ovarian pathology. Targeting this p300–SP1–BRD4 axis offers a promising strategy to restore ovarian function and opens new avenues for epigenetic therapy in PCOS.

## Ethics Statement

All animal procedures of the animal experiments and clinical data were approved by the Ethics Committee and the Institutional Animal Care and Use Committee of Nanjing Drum Tower Hospital, Nanjing University Medical School (2024AE01082).

## Consent

The author has nothing to report.

## Author Contributions

Conceptualization, G.J.T., W.S.C., Y.W., and D.J.W.; methodology, Z.Q.Z., Y.H.W., H.Y.C., and D.J.W.; investigation, Z.Q.Z., Y.H.W., X.Y.Y., T.Y.W., Y.J.W., and M.H.G., Y.H.; data analysis, Z.Q.Z., Y.H.W., H.Y.C., and D.J.W. writing – original draft, Z.Q.Z., W.S.C., and D.J.W.; writing – review & editing, G.J.T., W.S.C., Y.W., and D.J.W.; visualization, Z.Q.Z., W.S.C., Y.W., and D.J.W.; supervision, G.J.T., Y.W., and D.J.W.; funding acquisition, D.J.W and W.S.C.

## Conflicts of Interest

The authors declare no conflicts of interest.

## Supporting information




**Supporting File 1**: advs74396‐sup‐0001‐SuppMat.docx.

**Supporting File 2**: advs74396‐sup‐0002‐TableS1.docx

**Supporting File 3**: advs74396‐sup‐0003‐figureS1.jpg

## Data Availability

The data supporting the findings of this study are all available in the paper. Raw data of ATAC‐seq and RNA‐seq in this study are available from NCBI databases (PRJNA1254417 and PRJNA1255374). All data underlying this study are available from the corresponding author upon request.
