# Supplementary material for: Identification of A p300–SP1–BRD4 Transcriptional Axis as a Key Driver of AR Hyperactivation in Polycystic Ovarian Syndrome
Source: Adv Sci (Weinh). 2026 Feb 13;13(23):e18185. doi: 10.1002/advs.202518185 (PMC13104117; doi:10.1002/advs.202518185)
Supplement: Supplementary file 1 — Supporting File 1: advs74396‐sup‐0001‐SuppMat.docx. [file ADVS-13-e18185-s002.docx]

**Supplementary Table S1 Primers of the mouse and human genes for ChIP assay used in the study.**

| **Genes** | **Forward (5’ to 3’)** | **Reverse (5’ to 3’)** |
| --- | --- | --- |
| m*Ar* | GCCTTCAACCATACTACGC  (-187/-169) | GGGAGGTGGAAAGCAAA  (-16/1) |
| h*AR* | AGCACTTGTTTCTCCAAAGCCACT  (-163/-140) | GCCTCCTTGCCTTCCCACCT  (-77/-58) |


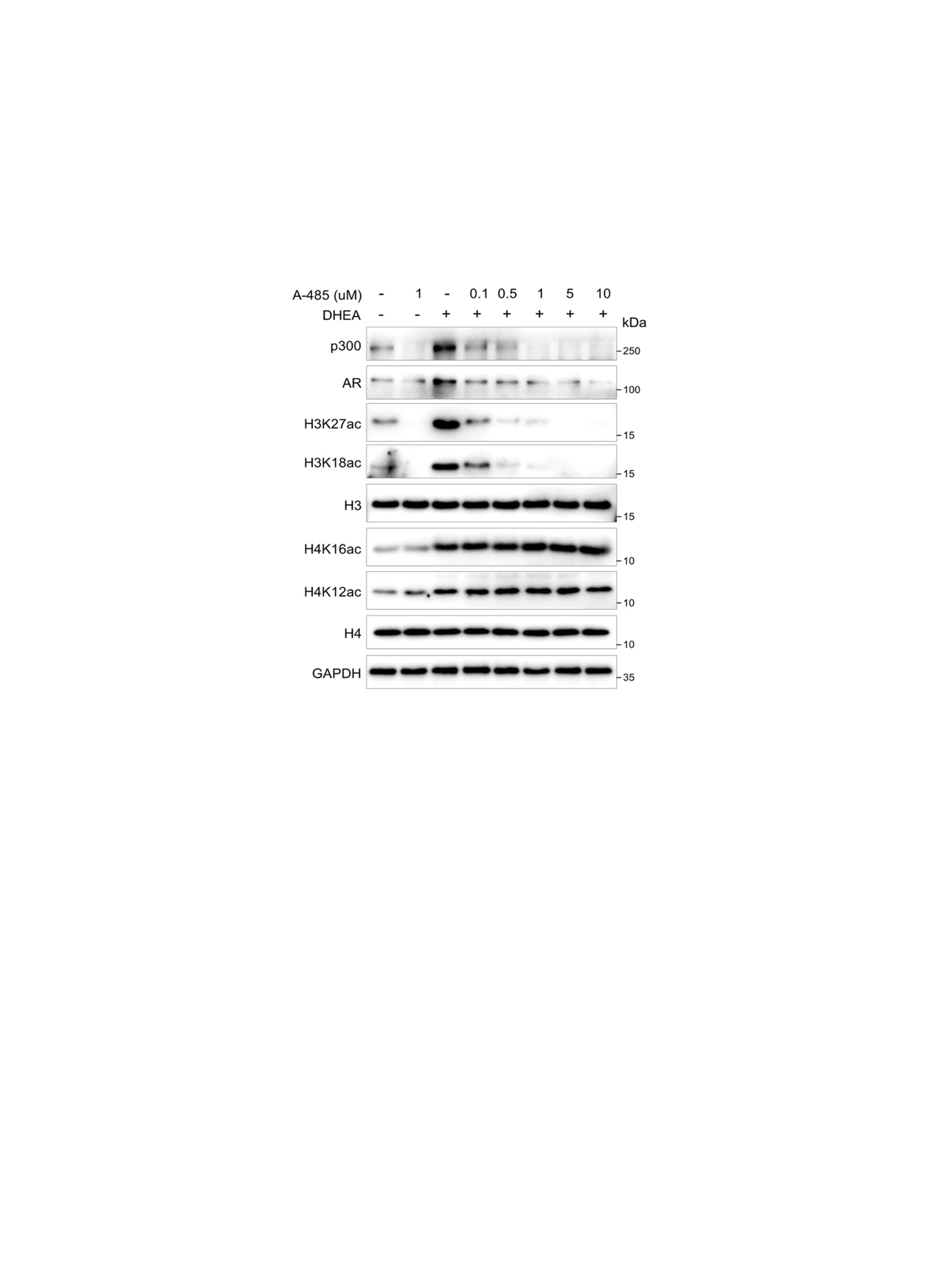


**Supplementary Figure1. The specific p300 inhibitor A-485 reversed the DHEA-induced upregulation of AR and the increase in H3K18ac and H3K27ac in primary GC cells.** GCs were treated with DHEA (25 μM, 48h) followed by varying concentrations of A-485 (0, 0.1, 0.5, 1, 5, 10 μM, 24h) treatment, and then cell lysates were assayed for p300, AR, H3K18ac, H3K27ac, H4K16ac, H4K12ac, H3, H4, and GAPDH.
