# Supplementary material for: Identification of A p300–SP1–BRD4 Transcriptional Axis as a Key Driver of AR Hyperactivation in Polycystic Ovarian Syndrome
Source: Adv Sci (Weinh). 2026 Feb 13;13(23):e18185. doi: 10.1002/advs.202518185 (PMC13104117; doi:10.1002/advs.202518185)
Supplement: Supplementary file 2 — Supporting File 2: advs74396‐sup‐0002‐TableS1.docx [file ADVS-13-e18185-s003.docx]

**Table S1 Primers of the mouse and human genes for ChIP assay used in the study.**

| **Genes** | **Forward (5’ to 3’)** | **Reverse (5’ to 3’)** |
| --- | --- | --- |
| m*Ar* | GCCTTCAACCATACTACGC  (-187/-169) | GGGAGGTGGAAAGCAAA  (-16/1) |
| h*AR* | AGCACTTGTTTCTCCAAAGCCACT  (-163/-140) | GCCTCCTTGCCTTCCCACCT  (-77/-58) |
