# Supplementary figures and images for: Identification of A p300–SP1–BRD4 Transcriptional Axis as a Key Driver of AR Hyperactivation in Polycystic Ovarian Syndrome
Source: Adv Sci (Weinh). 2026 Feb 13;13(23):e18185. doi: 10.1002/advs.202518185 (PMC13104117; doi:10.1002/advs.202518185)

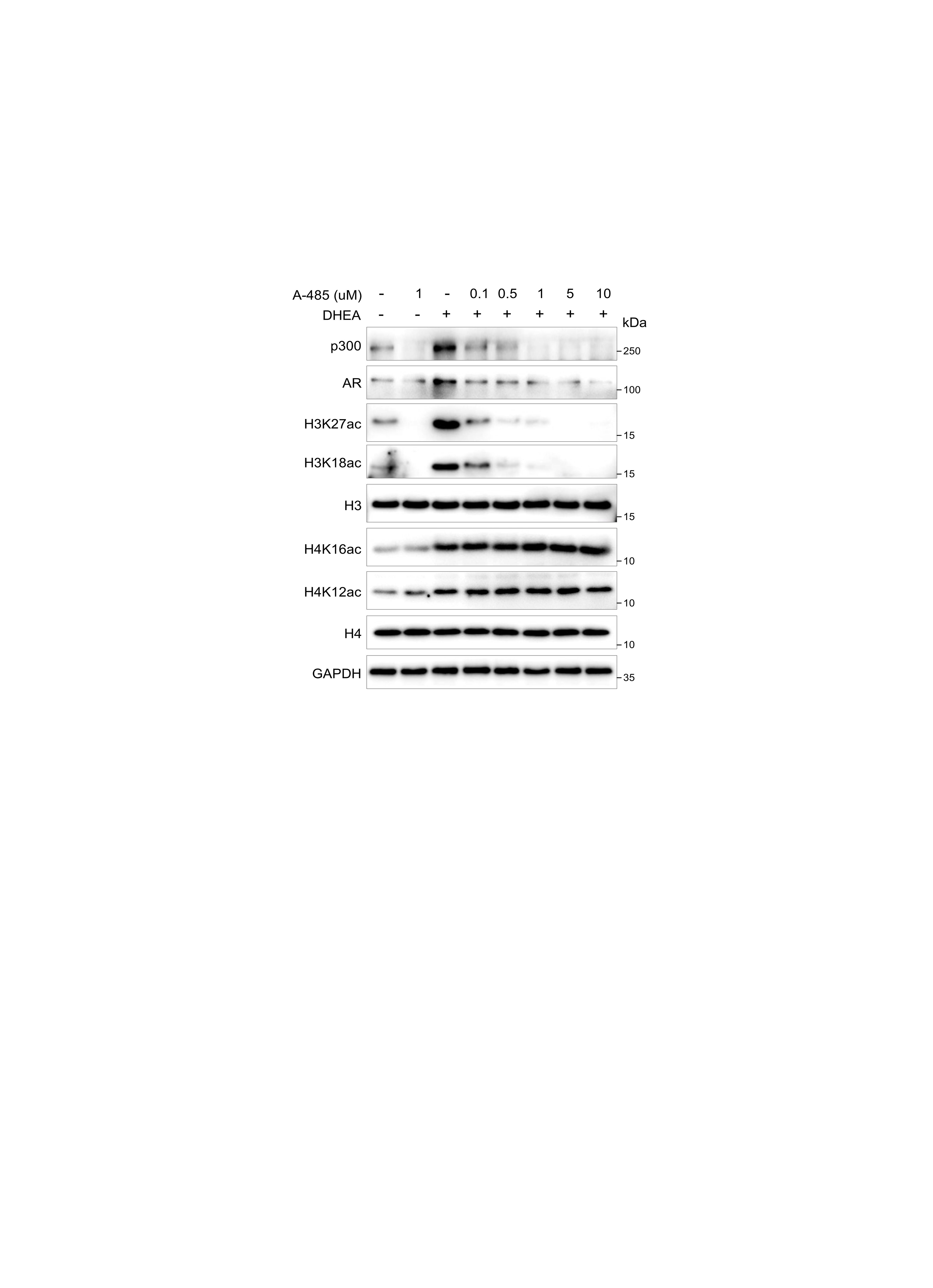

Supplement: Supplementary file 3 — Supporting File 3: advs74396‐sup‐0003‐figureS1.jpg [file ADVS-13-e18185-s001.jpg]
